# Predictive diagnosis of chronic obstructive pulmonary disease using serum metabolic biomarkers and least‐squares support vector machine

**DOI:** 10.1002/jcla.23641

**Published:** 2020-11-03

**Authors:** Hong Zheng, Yiran Hu, Li Dong, Qi Shu, Mingyang Zhu, Yuping Li, Chengshui Chen, Hongchang Gao, Li Yang

**Affiliations:** ^1^ Department of Pulmonary and Critical Care Medicine The First Affiliated Hospital of Wenzhou Medical University Wenzhou China; ^2^ Institute of Metabonomics & Medical NMR School of Pharmaceutical Sciences Wenzhou Medical University Wenzhou China

**Keywords:** artificial intelligence, COPD, diagnosis, lipoprotein, metabolomics

## Abstract

**Objective:**

Development of biofluid‐based biomarkers is attractive for the diagnosis of chronic obstructive pulmonary disease (COPD) but still lacking. Thus, here we aimed to identify serum metabolic biomarkers for the diagnosis of COPD.

**Methods:**

In this study, we investigated serum metabolic features between COPD patients (n = 54) and normal individuals (n = 74) using a ^1^H NMR‐based metabolomics approach and developed an integrated method of least‐squares support vector machine (LS‐SVM) and serum metabolic biomarkers to assist COPD diagnosis.

**Results:**

We observed a hypometabolic state in serum of COPD patients, as indicated by decreases in N‐acetyl‐glycoprotein (NAG), lipoprotein (LOP, mainly LDL/VLDL), polyunsaturated fatty acid (pUFA), glucose, alanine, leucine, histidine, valine, and lactate. Using an integrated method of multivariable and univariate analyses, NAG and LOP were identified as two important metabolites for distinguishing between COPD patients and controls. Subsequently, we developed a LS‐SVM classifier using these two markers and found that LS‐SVM classifiers with linear and polynomial kernels performed better than the classifier with RBF kernel. Linear and polynomial LS‐SVM classifiers can achieve the total accuracy rates of 80.77% and 84.62% and the AUC values of 0.87 and 0.90 for COPD diagnosis, respectively.

**Conclusions:**

This study suggests that artificial intelligence integrated with serum metabolic biomarkers has a great potential for auxiliary diagnosis of COPD.

## Graphical abstract

Development of biofluid‐based biomarkers is of great importance and interest for the diagnosis of chronic obstructive pulmonary disease (COPD) but still lacking. Here, we identified N‐acetyl‐glycoprotein (NAG) and lipoprotein (LOP) as two important metabolic biomarkers for distinguishing between COPD patients and healthy subjects. Then, we developed a LS‐SVM classifier based on NAG and LOP and found that LS‐SVM classifiers with linear and polynomial kernels performed better than the classifier with RBF kernel. Linear and polynomial LS‐SVM classifiers can achieve the total accuracy rates of 80.77% and 84.62% and the AUC values of 0.87 and 0.90 for COPD diagnosis, respectively. Thus, our study suggests that artificial intelligence with serum metabolic biomarkers might be a promising tool for auxiliary diagnosis of COPD.

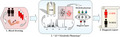

**Abbreviations**
COPD: chronic obstructive pulmonary disease
LOP: lipoprotein
LS‐SVM: least‐squares support vector machine
NAG: N‐acetyl‐glycoprotein
pUFA: polyunsaturated fatty acid

## 1 INTRODUCTION

Chronic obstructive pulmonary disease (COPD) is a preventable and treatable disease characterized by persistent respiratory symptoms and airflow limitation.^1^ Chronic obstructive pulmonary disease has become the third leading cause of death in the world and causes considerable economic and social burdens due to insufficient diagnosis and treatment.^2^, ^3^ Currently, spirometry is still a common method for diagnosing and monitoring progression of COPD according to the presence of chronic airflow limitation. Many factors may affect COPD diagnosis and lead to under‐ and over‐diagnosis^4^; therefore, it is of great importance to develop other adjunctive measures, especially biofluid‐based method. It is worth noting that serum inflammatory and oxidative stress markers have been associated with COPD.^5^, ^6^, ^7^, ^8^ However, there is still a lack of reliable and simple biofluid‐based biomarkers to assist the diagnosis of COPD.

Metabolomics has the ability to identify specific metabolic biomarkers related to the onset and development of disease,^9^ which makes it possible to diagnose or predict diseases, such as cancer,^10^ cardiovascular disease,^11^ and diabetes.^12^ Of note, characteristic metabolic changes have also been detected in COPD patients using a metabolomics approach. Ubhi et al found an increased protein turnover in serum of COPD patients by NMR‐based metabolomics.^13^ In exhaled breath condensate, COPD patients showed lower levels of acetone, valine, and lysine, as well as higher levels of lactate, acetate, propionate, serine, proline, and tyrosine, when compared with controls.^14^ Using a mass spectrometry‐based metabolomics method, Naz et al reported that oxidative stress and the autotoxin‐lysoPA axis were disturbed in serum of COPD patients in a sex‐specific manner.^15^

Additionally, artificial intelligence (AI)‐based techniques are developing rapidly in medicine and may achieve a better detection and diagnosis of disease.^16^, ^17^ For example, Esteva et al trained deep neural networks with skin images and achieved dermatologist‐level classification of skin diseases.^18^ Ardila et al developed a deep learning model integrated with computed tomography information to predict the risk of lung cancer with an area under the curve of 94.4%.^19^ Besides, AI‐based diagnostic technique has also been successfully applied for other diseases, such as liver masses,^20^ breast cancer metastases,^21^ diabetic retinopathy,^22^ and others. Most of the developed AI diagnostic systems are based on medical imaging; however, here we sought to integrate AI technique and metabolic features in biofluids for disease diagnosis and classification.

In the present study, therefore, we analyzed serum metabolic profiles in COPD patients and normal controls by using a ^1^H NMR‐based metabolomics approach. The aims of this study are (a) to identify characteristic metabolic changes in COPD patients, and (b) to develop an integrated method of least‐squares support vector machine and serum metabolomics biomarkers for auxiliary diagnosis of COPD.

## 2 MATERIALS AND METHODS

### 2.1 Clinical sample collection

We recruited a total of 128 participants from the First Affiliated Hospital of Wenzhou Medical University, including 54 COPD patients and 74 subjects without COPD. Pulmonary function was evaluated using prebronchodilator spirometry based on the Global Initiative for Chronic Obstructive Lung Disease (GOLD) criteria, and COPD was defined when FEV1 < 80% and FEV1/FVC < 0.7.^23^ The detailed clinical information of participants is listed in Table 1. Fasting blood sample was collected in a 5 ml vacutainer tube containing the chelating agent ethylene diamine tetraacetic acid (EDTA) and centrifuged at 1500 g for 15 minutes at 4°C. Serum was collected and stored at −80°C until analysis. This study was approved by the Ethical Committee of Wenzhou Medical University, and written informed consents were acquired from all subjects. All procedures in the present study were carried out according to the 2008 Helsinki Declaration and the clinical‐ethical guidelines of the First Affiliated Hospital of Wenzhou Medical University.

**Table 1:** The clinical information of participants

	COPD^a^	NORM^b^	*P*
n	54	74	–
Female, n (%)	7 (12.97)	43 (58.11)	–
Age (y)	71.27 ± 7.37	65.09 ± 7.89	<.001
Height (cm)	162.64 ± 8.36	157.06 ± 11.77	.007
Weigh (kg)	60.68 ± 9.88	63.03 ± 10.16	.22
Smoking status: current, n (%)	32 (59.26)	27 (36.49)	–
Smoking status: former, n (%)	23 (71.88)	12 (44.44)	–
Passive smoking status: never, n (%)	16 (29.63)	35 (47.30)	–
Passive smoking status: former, n (%)	10 (18.52)	9 (12.16)	–
Passive smoking status: current, n (%)	18 (33.33)	32 (43.24)	–
FVC^c^ (L)	2.3 ± 0.87	2.43 ± 0.69	.377
FVC (%)	74.25 ± 20.67	91.22 ± 19.76	<.001
FEV1^d^ (L)	1.28 ± 0.64	3.21 ± 10.45	.225
FEV1 (%)	51.2 ± 19.04	83.2 ± 21.11	<.001
FEV1/FVC (%)	53.67 ± 10.91	84.13 ± 6.76	<.001
Hypertension, n (%)	19 (35.19)	40 (54.05)	–
Diabetes, n (%)	4 (7.41)	5 (6.76)	–

^a^ Chronic obstructive pulmonary disease.

^b^ Participants without chronic obstructive pulmonary disease.

^c^ Forced vital capacity.

^d^ Forced expiratory volume in 1 s.

### 2.2 NMR‐based metabolomic analysis

^1^H NMR spectra were recorded using a Bruker AVANCE III 600 MHz NMR spectrometer with a 5‐mm TXI probe (Bruker BioSpin, Rheinstetten, Germany) at 37°C. Serum sample was thawed at 4°C and vortexed for 10 seconds using a vortex‐genie (Scientific Industries). Then 200 μL of serum sample was drawn into an Eppendorf tube and mixed with 400 μL of 0.2 mol/L phosphate buffer. The mixture was centrifuged at 10 000 g for 10 minutes at 4°C, and 500 μL of supernatant was transferred and mixed with 100 μL of D_2_O containing 0.5% sodium trimethylsilyl propionate‐d_4_ (TSP) in a 5 mm NMR tube for metabolomics analysis. ^1^H NMR spectra were acquired using the CPMG pulse sequence with a fixed receiver‐gain value and the main parameters were set as follows: relaxation delay, 4 seconds; acquisition time, 1.64 seconds/scan; data points, 32K; spectral width, 10 000 Hz; exponential line‐broadening function, 0.3 Hz.

All NMR spectra were phase/baseline corrected automatically and referenced to the methyl signal of lactate at 1.33 ppm in Topspin 3.0 software (Bruker BioSpin). Subsequently, all spectra were aligned using the “icoshift” procedure in MATLAB (R2012a, The Mathworks Inc).^24^ NMR spectra from 0.4 to 9.0 ppm excluding the residual water region from 4.0 to 5.0 ppm were subdivided and integrated to binning data with a size of 0.01 ppm for multivariate analysis.

Metabolite signals in NMR spectra were assigned by using Chenomx NMR suite 7.0 (Chenomx Inc) and the human metabolome database.^25^ To further confirm uncertain identifications, a two‐dimensional ^13^C‐^1^H heteronuclear single quantum coherence (HSQC) experiment was employed to analyze the representative samples. The level of each metabolite was indicated using its peak area.

### 2.3 Multivariate data analysis

Partial least‐squares‐discriminate analysis (PLS‐DA) was performed on auto‐scaled data to obtain an overview of metabolic changes between COPD patients and normal individuals by using MetaboAnalyst 4.0.^26^ Moreover, a permutation test with 1,000 permutations based on separation distance was used to validate the performance of PLS‐DA models.^26^ In PLS‐DA, variable importance in the projection (VIP) represents a quantitative statistical parameter ranking metabolites according to their ability to discriminate between COPD patients and normal individuals. In this study, metabolites with VIP values more than 1.5 were selected as important indicators.

### 2.4 Least‐squares support vector machine (LS‐SVM) classifier

Least‐squares support vector machine as an artificial intelligence model was used to distinguish COPD patients from normal individuals. For development of LS‐SVM classifier, the selection of optimal kernels and parameters is crucial for model performances. Therefore, in the present study, linear, polynomial and RBF kernels were compared, and leave one out cross‐validation were used to select the optimal parameters of LS‐SVM. All data were auto‐scaled and randomly divided into two subsets for training (80%) and testing (20%) phases of LS‐SVM classifiers. Receiver operating‐characteristic (ROC) curve was plotted with sensitivity versus 1‐specificity, and its area under curve (AUC) value was calculated to assess the diagnostic performance of LS‐SVM classifiers. LS‐SVM model with the highest AUC value was selected as the optimal model for COPD predictive diagnosis. LS‐SVM was implemented using a LS‐SVM toolbox^27^ under MATLAB environment (R2012a, The MathWorks, Inc, Natick, MA, USA).

### 2.5 Statistical analysis

Metabolic difference between COPD patients and normal individuals was performed using Student's *t* test with Bonferroni correction in SAS software (SAS 9.2, SAS Institute Inc), and a statistically significant difference was defined when a *P* value below .05. The volcano plot was employed to identify potentially important metabolic markers according to fold change and P value of the metabolite using MetaboAnalyst 4.0.^26^ The AUC value of each metabolite for COPD predictive diagnosis was calculated by a classical univariate ROC analysis in MetaboAnalyst 4.0.^26^

## 3 RESULTS

### 3.1 COPD patients possesses a peculiar metabolic phenotype

**FIGURE 1:**
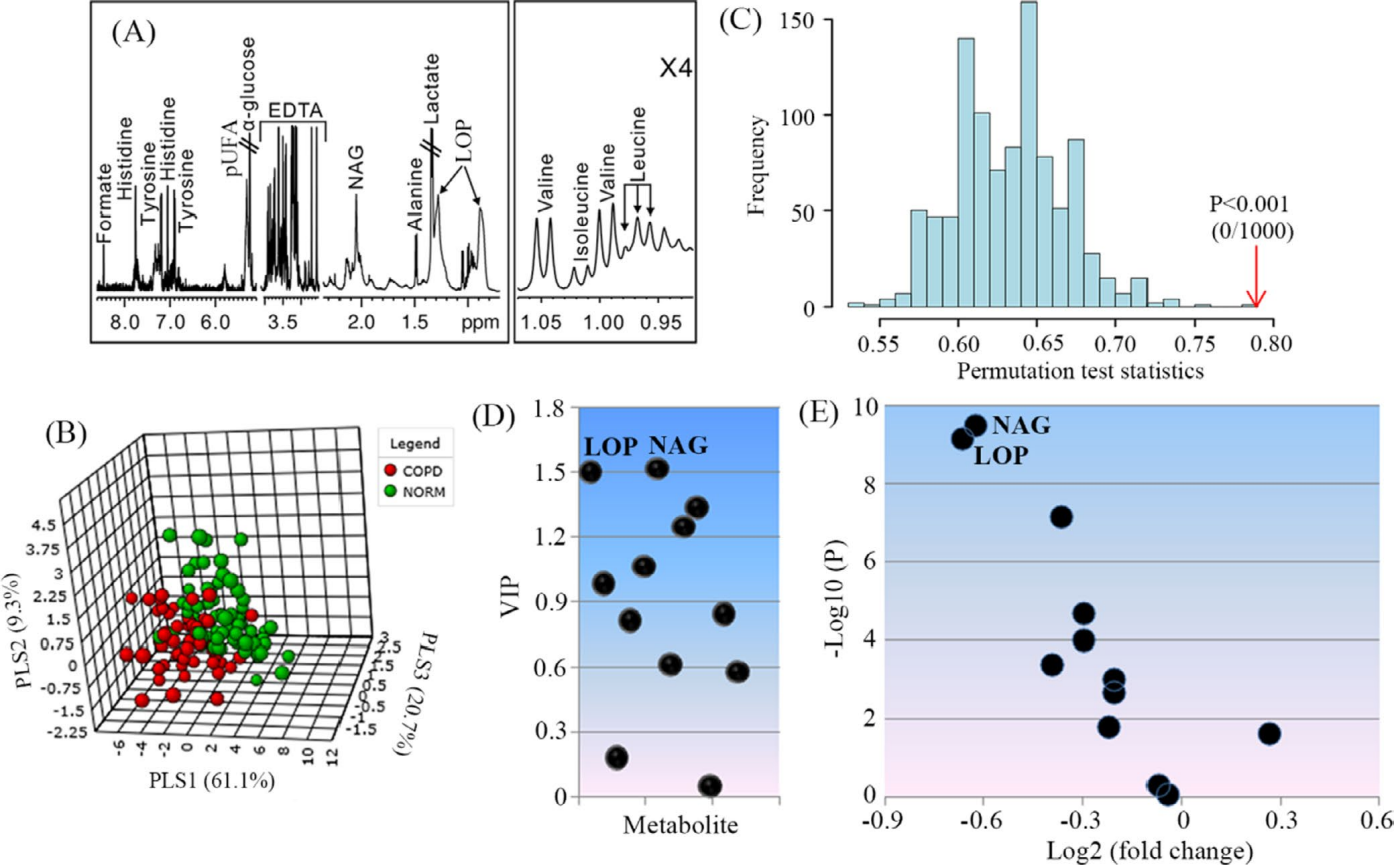
NMR‐based metabolomics analysis. A, A typical 600 MHz ^1^H NMR spectrum obtained from serum in patients with COPD: LOP, lipoprotein; NAG, N‐acetyl‐glycoprotein; ×4, 4 times magnification; B, PLS‐DA‐based classification of participators with and without COPD (COPD vs. NORM) using serum metabolomics data; C, A 1000‐times random permutation test; D, VIP values of metabolites analyzed by PLS‐DA; E, Volcano plot of metabolites analyzed by MetaboAnalyst. Important metabolites were selected when VIP value was >1.5 and *P* value <.05

Typical ^1^H NMR spectrum acquired from serum in COPD patients is illustrated in Figure 1A, where we identified a series of serum metabolites, involving amino acid metabolism (alanine, valine, isoleucine, leucine, tyrosine, and histidine), glucose metabolism (lactate and glucose), lipid metabolism (pUFA, polyunsaturated fatty acid; formate), and others (LOP, lipoprotein mainly include LDL and VLDL; NAG, N‐acetyl‐glycoprotein). To examine the difference of metabolic patterns between COPD patients and controls, a partial least‐squares‐discriminant analysis (PLS‐DA) was used in this study based on serum metabolomes, as shown in Figure 1B. The result of PLS‐DA shows a clear separation between COPD patients and controls. Then, we performed a permutation test with 1000 random permutations to test this model and found a statistically significant performance of PLS‐DA model (Figure 1C, *P* < .001). Figure 1D shows variable importance in projection (VIP) scores of each metabolite from PLS‐DA model. Relative to other metabolites, NAG and LOP with VIP > 1.5 were identified as important differential metabolites between COPD patients and controls in this study. Moreover, volcano plot also shows that NAG and LOP had a higher fold change and more significant difference (Figure 1E).

**FIGURE 2:**
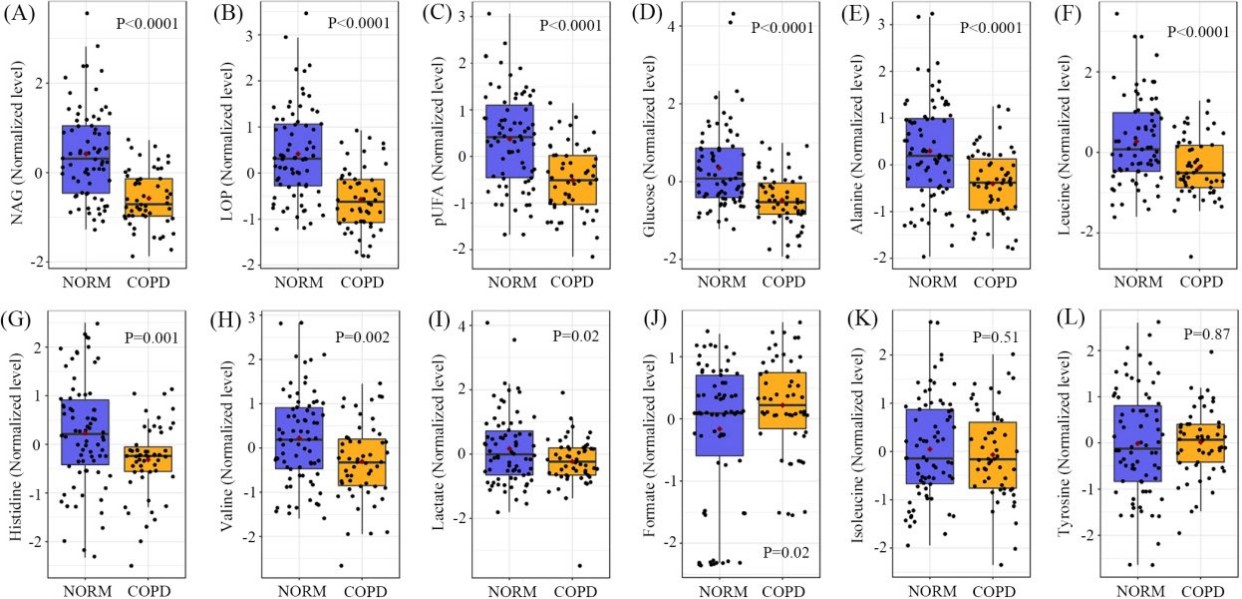
COPD‐driven serum metabolic changes. The metabolic difference between COPD patients (COPD) and normal individuals (NORM) was analyzed using Student's *t* test with Bonferroni correction, and a statistically significant difference was defined when a *P* value < .05. Metabolite: pUFA, polyunsaturated fatty acid; LOP, lipoprotein; NAG, N‐acetyl‐glycoprotein

Furthermore, we found that most of identified metabolites in serum were significantly decreased in COPD patients relative to normal controls, including NAG (Figure 2A), LOP (Figure 2B), pUFA (Figure 2C), glucose (Figure 2D), alanine (Figure 2E), leucine (Figure 2F), histidine (Figure 2G), valine (Figure 2H), and lactate (Figure 2I). However, COPD patients had a significantly higher level of serum formate than controls (Figure 2J, *P* = .02). In addition, there were no significant differences in serum isoleucine (Figure 2K, *P* = .51) and tyrosine (Figure 2L, *P* = .87) levels between COPD patients and controls.

### 3.2 Diagnosis of COPD based on LS‐SVM classifier using metabolic biomarkers

**FIGURE 3:**
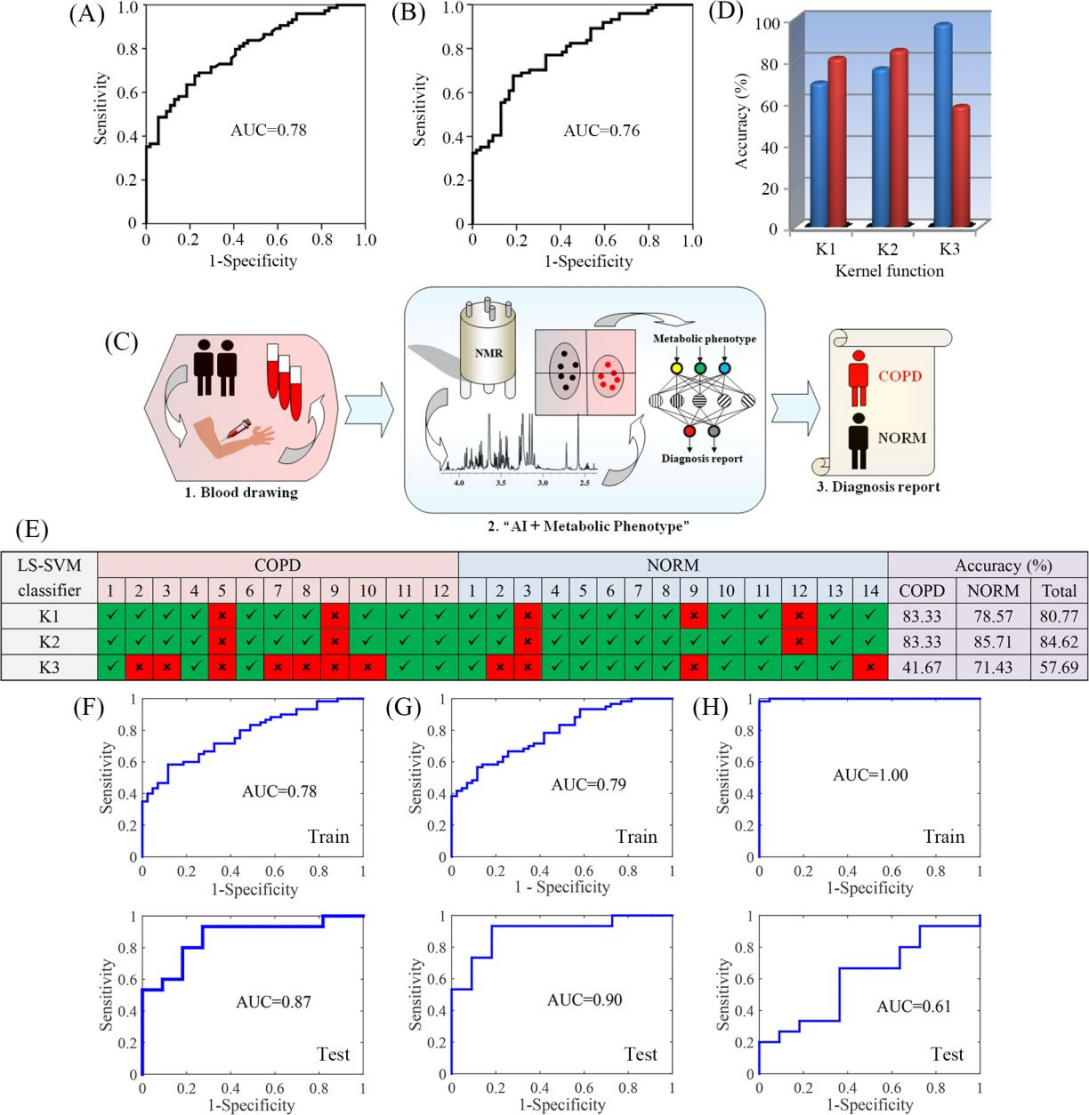
AI‐based COPD diagnosis using serum metabolic biomarkers. Values of the area under the curve of (A) N‐acetyl‐glycoprotein (NAG) and (B) lipoprotein (LOP) analyzed by receiver operating characteristics (ROC); (C) AI‐based diagnostic procedure of COPD: (1) Serum collection, (2) Predictive diagnosis of COPD based on AI and metabolic biomarkers, and (3) Diagnosis report; (D) The diagnostic accuracies of COPD using LS‐SVM classifiers equipped with different kernel functions and serum metabolic biomarkers (NAG and LOP): K1, linear function; K2, polynomial function; K3, radial basis function; (E) The diagnostic results using LS‐SVM with different kernel functions and serum metabolic biomarkers based on an independent dataset including 12 COPD patients and 14 subjects without COPD (NORM); the ROC analyses and AUC values of LS‐SVM with (F) linear, (G) polynomial, and (H) radial basis functions during the training and test phases

In this study, ROC curves analysis was employed to evaluate serum NAG and LOP that have been identified as important differential metabolites for diagnosis of COPD. The corresponding area under curve (AUC) were 0.78 for NAG (Figure 3A) and 0.76 for LOP (Figure 3B). Subsequently, we developed LS‐SVM classifiers equipped with different kernel functions for COPD diagnosis using NAG and LOP, as shown in Figure 3C. The development of LS‐SVM classifier includes two steps: training and test phases. In the training phase, 80% of the data were randomly selected to generate the models. We found that LS‐SVM classifier with radial basis function (RBF) kernel had a higher accuracy (Figure 3D) than the classifier with other two kernels. After training, LS‐SVM classifiers were tested using an independent dataset (20% of the data). The results reveal that the total classification accuracy of LS‐SVM classifier with RBF kernel were dramatically decreased to 57.69% (Figure 3E), suggesting that this model was overfitting. During the test phase, we found that the classification accuracy of LS‐SVM classifier with RBF kernel was only 41.67% for COPD patients and 71.43% for normal controls. For linear LS‐SVM classifier, however, the classification accuracies were 83.33% and 78.57% for COPD patients and normal controls, respectively, and the total accuracy rate was 80.77%, as shown in Figure 3E. For polynomial LS‐SVM classifier, the classification accuracy was 83.33% for COPD patients and 85.71% for normal controls, and the total accuracy rate was 84.62% (Figure 3E). In addition, linear (Figure 3F) and polynomial (Figure 3G) LS‐SVM classifiers achieved the AUC values of 0.87 and 0.90 for COPD diagnosis in an independent dataset, respectively; however, the AUC value of LS‐SVM classifier with RBF kernel was only 0.61 (Figure 3H). Thus, for the diagnosis of COPD based on NAG and LOP, LS‐SVM classifiers with linear and polynomial kernels performed better than the classifier with RBF kernel.

## 4 DISCUSSION

Abnormal metabolism plays an important role in most diseases, indicating that the onset and development of diseases would be accompanied by a peculiar metabolic change.^28^ Hence, metabolomics might be contributed to explore the pathogenesis and treatment of diseases as well as to predict and diagnose diseases. In the present study, we observed a hypometabolic state in COPD patients using an NMR‐based metabolomics approach. This finding is consistent with the result of Labaki et al, who reported that the severity of airflow obstruction is linked with downregulation of serum metabolism in smokers.^29^ In addition, they also identified the most relevant metabolites, including tryptophan, histidine, valine and leucine.^29^ Therefore, hypometabolism may trigger the progression of COPD.

In this study, we found that COPD patients had lower leucine and valine levels than normal controls. Leucine and valine belong to branched‐chain amino acids (BCAAs) that have been shown to regulate protein turnover and glucose homeostasis.^30^ Decreased BCAAs levels in COPD patients have also been reported in previous studies.^31^, ^32^ Additionally, Yoneda et al demonstrated that reduced BCAAs levels in COPD patients are specifically related to loss of body weight and muscle mass.^33^ Besides BCAAs, we also observed significantly decreased levels of alanine and histidine in serum of COPD patients relative to normal controls. For COPD, cachexia is regarded as a common and partly reversible feature, but adversely affects its progression and prognosis.^34^, ^35^ Of note, amino acids have been shown to be implicated in COPD cachexia.^32^, ^35^ In the body, amino acids not only are necessary constituents for protein synthesis, but also replenish tricarboxylic acid (TCA) cycle intermediates for energy supply.^36^ Therefore, the reduction of amino acid metabolism could be a common characteristic in COPD patients and indicate the deterioration of COPD.

Chronic obstructive pulmonary disease has also been associated with disrupted lipid metabolism,^37^, ^38^ but its effect is differed on blood lipid profiles. Titz et al reported that smokers with COPD can be clearly distinguished from never‐smokers using serum lipid profiles.^39^ They also found that COPD patients had higher levels of glycerolipids and monounsaturated fatty acids as well as lower levels of polyunsaturated fatty acids (pUFA) and hydroxyoctadecadienoic acids.^39^ In addition, decreased pUFA level in COPD patients was also detected in other studies.^40^, ^41^, ^42^ In the current study, relative to normal controls, we observed a lower pUFA level and a higher formate level in serum of COPD patients. Of note, a decrease in pUFA is a marker of oxidative stress,^43^, ^44^ and an increase in formate has been implicated in the inflammatory process.^45^ Therefore, changes in lipid metabolism proposed herein may indicate increases in oxidative stress and inflammation in COPD patients compared with normal controls, which could be potential inducements for COPD progression. Additionally, glucose metabolism was also vulnerable to be disturbed in COPD patients relative to normal individuals.^46^ In this study, we found that COPD patients had significantly lower levels of glucose and lactate in serum than normal controls. This finding suggests an impaired energy metabolism in COPD patients.^47^, ^48^ Together, our results imply that the disturbance of glucose and lipid metabolism could be one of main causes in COPD.

We speculate that the downregulation of amino acid, glucose and lipid metabolism would result in decreases in glycoprotein and lipoprotein. As expected, our data show that COPD patients had significantly lower levels of NAG (N‐acetyl‐glycoprotein) and LOP (mainly LDL and VLDL) in serum than normal controls. In our study, NAG and LOP were also identified as two important metabolites for distinguishing between COPD patients and controls from both multivariable and univariate analyses. Therefore, we sought to develop an artificial intelligence (AI) model using these two potential biomarkers for predictive diagnosis of COPD. AI‐based diagnostic approach has been used in COPD. For example, Fernandez‐Granero et al developed a decision tree classifier based on respiratory sounds of patients to early predict COPD exacerbation.^49^ Feature‐weighted survival learning machine using all risk factors in medical records was established for COPD failure prediction.^50^ Additionally, Goto et al also reported that machine learning approaches can predict disposition of COPD exacerbation.^51^ In the present study, LS‐SVM classifiers equipped with linear and polynomial kernels based on serum NAG and LOP levels can achieve the total accuracy rates of 80.77% and 84.62% and the AUC values of 0.87 and 0.90 for COPD diagnosis, respectively. Our results demonstrated that an integrated method of AI technique and biofluid biomarkers has a significant potential for auxiliary diagnosis of COPD.

## 5 CONCLUSIONS

We used NMR‐based serum metabolomics to examine metabolic differences between COPD and normal individuals and detected a hypometabolic state in COPD patients. The peculiar metabolic phenotype of COPD mainly included the decreases in amino acid, glucose, and lipid metabolism. Moreover, we identified NAG and LOP as two important metabolites for distinguishing between COPD patients and normal individuals. LS‐SVM classifiers based on NAG and LOP were developed for COPD diagnosis, and LS‐SVM classifiers with linear and polynomial kernels performed better than the classifier with RBF kernel. The highlight of the present study is to develop an integrated method of artificial intelligence and biofluid‐based biomarkers for auxiliary diagnosis of COPD. However, the proposed diagnostic method still needs to be further validated in a large clinical sample.

## AUTHOR CONTRIBUTIONS

CCS, YPL, LY, and HCG contributed to the experimental design. LY, YRH, LD, and YPL contributed to clinical diagnosis and sample collection. QS, MYZ, and HZ contributed to NMR‐based metabolomics analysis. HZ contributed to the data analysis, result interpretation, and writing. All authors have reviewed and approved the final manuscript.
